# Association between poor blood pressure control and intracranial haemorrhage-related mortality in patients with atrial fibrillation receiving oral anticoagulation: a retrospective observational cohort study

**DOI:** 10.3389/fstro.2026.1840317

**Published:** 2026-08-14

**Authors:** Jin Un Kim, Zhen Cahilog, Zehra Kadani, Teledalase Olajoyegbe, Darren Fernandes, Dhiraj Ail, Karishma Oojageer, Telma Da Silva, Saeedur Rahman, Peter Kabunga

**Affiliations:** 1 Department of Cardiology, Darent Valley Hospital, Dartford, Kent, United Kingdom; 2 Darent Valley Hospital, Dartford, Kent, United Kingdom; 3 Department of Stroke Medicine, Darent Valley Hospital, Dartford, Kent, United Kingdom; 4 Department of Cardiology, King’s College Hospital, Denmark Hill, London, United Kingdom

**Keywords:** anticoagulation, atrial fibrillation, hypertension, hypertensive vasculopathy, intracranial haemorrhage (ICH)

## Abstract

**Study objective:**

Hypertension is a major modifiable risk factor for intracranial haemorrhage (ICH) in patients with atrial fibrillation (AF) receiving oral anticoagulation (OAC). We performed a retrospective exploratory analysis of the prevalence of poorly controlled hypertension, as evidenced by hypertensive vasculopathy on neuroimaging, among patients with AF on OAC who died after presenting with ICH at a large district general hospital in England.

**Methods:**

Between December 2020 and April 2024, the death registry data of a large district general hospital in the South East of England, United Kingdom, were analysed to identify patients who were anticoagulated for AF and who had ICH as the primary cause of their death. The confirmatory neuroimaging data were interrogated to assess the likely underlying cause of bleeding.

**Results:**

Of the 5,452 deaths within the study period, 46 patients on OAC for AF were included. The majority of patients (78.3%) had spontaneous ICH compared to traumatic ICH (11.7%). Most patients (84.8%) recorded hypertension (80% in the traumatic group, *n* = 8 vs. 86.1% in spontaneous ICH, *n* = 31). In those with spontaneous ICH, 28 (77.8%) demonstrated neuroradiological evidence of bleeding as a result of hypertensive vasculopathy.

**Conclusion:**

Hypertension is not only a major substrate of AF incidence but a cause of additional morbidity and mortality. Within our cohort, hypertensive vasculopathy was a significant underlying aetiology of spontaneous ICH-related mortality. Falls and trauma causing ICH in anticoagulated patients with AF may be overestimated. In line with the recently updated European Society of Cardiology (ESC) guideline, blood pressure should take priority in the management of AF to reduce major bleeding risk.

## 1 Introduction

Hypertension and atrial fibrillation (AF) often coexist and are among the most common cardiovascular comorbidities found in older patients (Kornej et al., 2020). The prevalence of hypertension in AF is approximately 60–80% and increases further with age (Nabauer et al., 2009). It is an important modifiable risk factor in AF; a 5 mmHg reduction in systolic blood pressure (BP) reduces the risk of major cardiovascular events by 9% (Pinho-Gomes et al., 2021). Hypertension independently increases the risk of stroke and bleeding, and therefore, stringent BP control is highlighted as a primary aim in the recently updated European guidelines for AF management (Friberg et al., 2012; Van Gelder et al., 2024).

Older patients with AF are commonly frail and multimorbid. They have worse clinical outcomes from ischaemic strokes but are often the subgroup that is inadequately treated with oral anticoagulant therapy (OAC) (Miyasaka et al., 2006; Wolf et al., 1991; Madhavan et al., 2019). A major barrier to withholding or withdrawing OAC relates to the risk of fatal intracranial haemorrhage (ICH), a major contributor to stroke-related mortality and dependency, in those who may be susceptible to excessive falls, dementia, or polypharmacy (de Almeida et al., 2020; Grymonprez et al., 2020). One study found that documented high falls risk in patients was associated with 1.9 times higher ICH risk, yet despite this, the benefit from OAC remained the same regardless of falls history (Gage et al., 2005). Furthermore, studies reveal that anticoagulated AF patients’ bleeding risk only becomes relevant if their falls frequency approximates 45–458 times annually (Man-Son-Hing et al., 1999; Wei et al., 2021).

Despite reassurances, spontaneous ICH unrelated to trauma remains a potentially fatal consequence, and the proportion of the causative factor being driven by OAC or by other mechanisms is not well established. Hypertensive vasculopathy is the commonest cause of spontaneous ICH (Kelly and Rothwell, 2020). As such, we sought to evaluate the death registry of AF patients on OAC who died of ICH to assess the presence of unmanaged risk factors and identify the proportion of hypertensive vasculopathy in those who presented with or without a trauma-related history.

## 2 Materials and methods

A retrospective review of the data from the Medical Examiner database at a large district general hospital in southeast England was undertaken to identify consecutive adult patients with atrial fibrillation (AF) receiving oral anticoagulation (OAC) whose Medical Certificate of Cause of Death (MCCD) listed intracranial haemorrhage (ICH) as a cause of death between December 2020 and April 2024. The Medical Examiner Service forms part of the statutory death certification process in the United Kingdom and supports completion of the MCCD in accordance with national regulations.

The cause of death was determined according to standard UK clinical practice. The treating clinician completed the initial MCCD, which was subsequently reviewed and confirmed by an independent Medical Examiner following scrutiny of the hospital records. Cases in which the cause of death remained uncertain were referred to the Coroner’s Office in accordance with national procedures.

Eligible patients were aged ≥18 years, had a documented diagnosis of AF, were receiving OAC at the time of ICH, and had ICH recorded as a cause of death on the MCCD. Attribution of ICH as the cause of death was corroborated using clinical records, neuroimaging, and post-mortem findings where available. Patients were excluded if they were not receiving OAC at the time of ICH, had no prior diagnosis of AF, died from a cause unrelated to ICH, or had insufficient clinical records to ascertain the study variables.

Demographic, clinical, medication, blood pressure, laboratory, imaging, and outcome data were extracted retrospectively from the electronic health record and associated hospital databases using a standardised data collection protocol.

Data on risk factors for thromboembolic events and major bleeding as indicated by the CHA2DS2-VASc and HAS-BLED scoring criteria were collected, and SPARCtool (version 10.1, http://www.sparctool.com) was used to calculate the net clinical benefit in relation to the patient’s specific mode of anticoagulation, annual risk of thromboembolism and major bleeding risk. Relevant computed tomography (CT) neuroimaging was interrogated by a radiologist and stroke physician to determine the likely ICH aetiology (Supplementary Figures 1–3). This was correlated with the MCCD and clinical documentation. Exclusion criteria included: no neuroimaging performed, erroneous initial neuroimaging report indicated by subsequent addendum, long duration between ICH and death, and ICH secondary to haemorrhagic transformation of ischaemic cerebrovascular accidents (CVA) or bleed from metastatic brain lesions.

Continuous variables were compared using the Mann–Whitney U-test due to non-normal distribution. Categorical variables were analysed using Fisher’s exact test given the small sample size. A two-tailed *p*-value of <0.05 was considered statistically significant.

This study was an exploratory retrospective analysis of all eligible cases within the defined study period; therefore, no *a priori* sample size calculation was performed. A *post hoc* estimation was undertaken to assess the robustness of the sample size. Assuming a prevalence of hypertensive vasculopathy of approximately 70–80% in spontaneous intracranial haemorrhage, a sample size of 36 patients provides an estimated precision of ±15% around the proportion at a 95% confidence level. The study is therefore adequately powered for descriptive analyses but may be underpowered to detect small between-group differences.

## 3 Results

**Figure 1:**
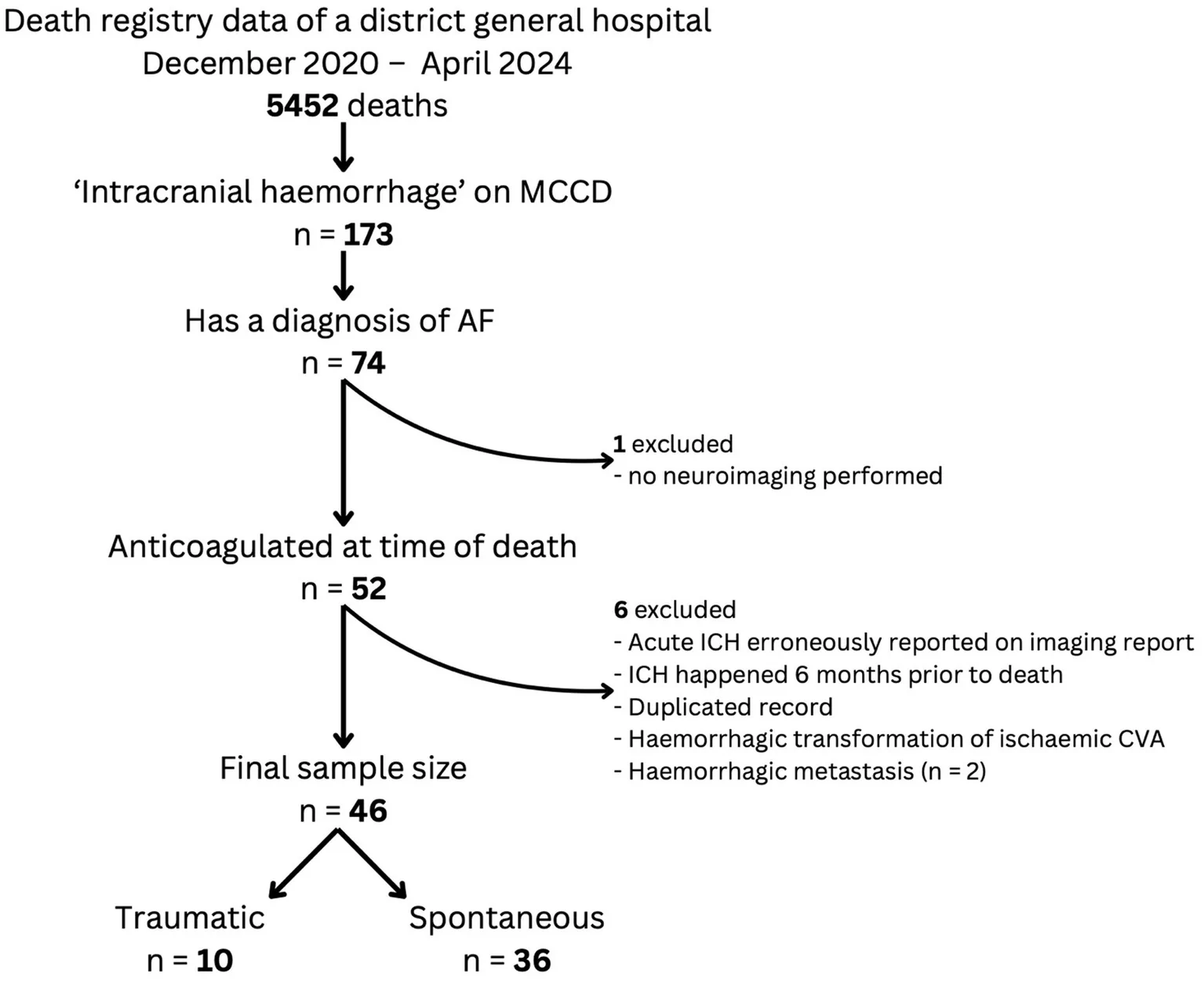
Flow diagram of participant selection. AF, atrial fibrillation; ICH, intracranial haemorrhage; CVA, cerebrovascular accident.

Of the 5,452 deaths registered between December 2020 and April 2024, 173 had ICH as a cause on the MCCD. A diagnosis of AF was identified in 74, and of these, 52 were anticoagulated. The final sample size after exclusion according to the aforementioned criteria was 46 (Figure 1).

### 3.1 Baseline characteristics and comorbidities

Table 1 shows an almost equal distribution between females (48%) and males (52%) in the study population, with a comparable median and distribution of age between the traumatic and spontaneous ICH cohorts (84.5 [83–88.3] vs. 83.5 [78.8–88.3], respectively) (Table 1). The ethnicity of the majority of the study population was identified as ‘British, Mixed British’ (95.7%). Most did not have an admission due to falls in the 12 months prior to death. One patient in the traumatic ICH group was excluded from this analysis due to no information available on previous admission or from GP records, precluding an accurate CHA2DS2-VA score. The median and distribution of the Clinical Frailty Scale (CFS) were similar between the traumatic and spontaneous ICH cohorts (5.5 [4.3–6.8] vs. 5 (), respectively). The majority of the study population had a diagnosis of hypertension (80% of the traumatic ICH cohort vs. 86.1% in the spontaneous ICH cohort). The second most prevalent comorbidity was a previous history of CVA (30% of the traumatic ICH cohort vs. 41.7% of the spontaneous ICH cohort). A history of major bleeding was exclusively seen in the spontaneous ICH cohort (8.3%).

**Table 1:** Baseline characteristics and comorbidities of the study population.

		Traumatic (*n* = 10)	Spontaneous (*n* = 36)	Total (*n* = 46)	*p*-value
Age (Median [IQR])		84.5 [83–88.3]	83.5 [78.8–88.3]		0.78
Sex (%)	Female	4 (40.0%)	18 (50.0%)	22 (47.8%)	0.72
Clinical Frailty Scale (Median [IQR])		5.5 [4.3–6.8]	5 [4–6]		0.65
Comorbidities (%)					
Congestive heart failure		2 (20.0%)	9 (25.0%)	11 (23.9%)	1.0
Hypertension		8 (80.0%)	31 (86.1%)	39 (84.8%)	0.64
Diabetes		4 (40.0%)	7 (19.4%)	11 (23.9%)	0.21
Vascular disease		2 (20.0%)	9 (25.0%)	11 (23.9%)	1.0
Prior TIA/Stroke		3 (30.0%)	15 (41.7%)	18 (39.1%)	0.72
Abnormal renal function (Cr > 200, transplant, dialysis)		1 (10.0%)	1 (2.8%)	2 (4.3%)	0.41
Abnormal liver function		1 (10.0%)	0*	1 (2.2%)	0.22
History of major bleeding		0	3 (8.3%)	3 (6.5%)	0.55
History of labile INR		0	1 (2.8%)	1 (2.2%)	1.0
Alcohol use		0	2 (5.6%)	2 (4.3%)	0.53
Type of anticoagulation (%)					
Warfarin		1 (10.0%)	4 (11.1%)	5 (10.9%)	1.0
Edoxaban		3 (30.0%)	6 (16.7%)	9 (19.6%)	0.38
Rivaroxaban		2 (20.0%)	8 (22.2%)	10 (21.7%)	1.0
Apixaban		4 (40.0%)	18 (50.0%)	22 (47.8%)	0.72
INR (Median [IQR])		1.7 [1.3–2]	1.4 [1.2–1.7]		0.32
Concomitant antiplatelet use (%)					
Aspirin		0	1 (2.8%)	1 (2.2%)	1.0
Clopidogrel		0	1 (2.8%)	1 (2.2%)	1.0

*INR result was unavailable for 1 patient in the ‘Spontaneous bleed’ group.

TIA, Transient Ischaemic Attack; INR, International Normalised Ratio; IQR, Inter-quartile range.

### 3.2 ICH characteristics and anticoagulation risk scoring

**Figure 2:**
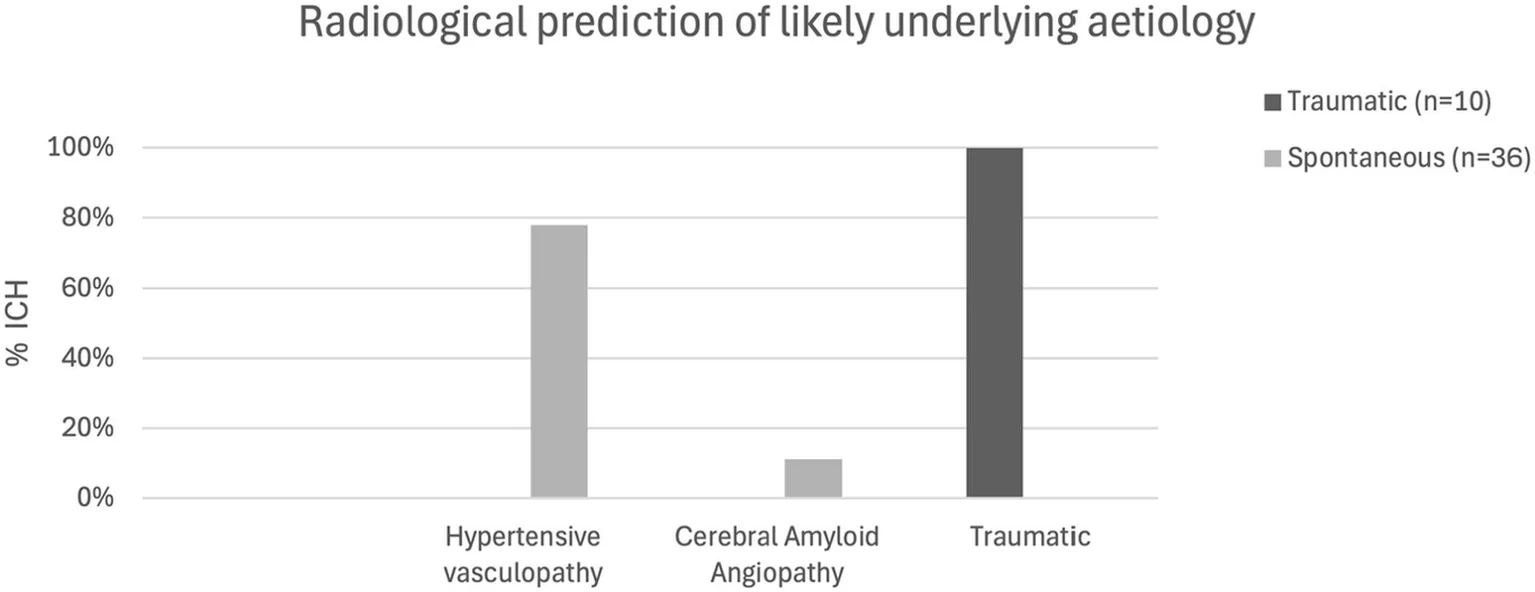
Radiological prediction by independent neuroradiologist of likely underlying aetiology for each type of ICH (stated on the Medical Certificate of Cause of Death). ICH, Intracranial haemorrhage.

In the traumatic ICH cohort, the predominant type of ICH identified from neuroimaging was subdural (80%), whereas most in the spontaneous ICH cohort had parenchymal haemorrhage with ventricular extension (66.7%). Hypertensive vasculopathy was the likely underlying cause of the ICH in most of the spontaneous group (77.8%), followed by cerebral amyloid angiopathy (11.1%) (Figure 2). The majority of the study population had conservative management of the ICH; only one underwent neurosurgical intervention.

There was an overall similar proportion of warfarin and OAC use, as well as the type of OAC used, between the traumatic and spontaneous ICH cohorts, with greater use of edoxaban in the traumatic group (30% vs. 16.7%). Most were not on concomitant antiplatelet therapy (95.7%) and had a similar median INR. Most of the study population had a normal range platelet count. Median CHA2DS2-VASc and CHA2DS2-VA scores were identical between the traumatic and spontaneous ICH cohorts (5 and 4.5, respectively) with a similar distribution, which was reflective of the annual thromboembolism risk with and without anticoagulation, as calculated by SPARCtool (Table 2).

**Table 2:** Intracranial haemorrhage (ICH) characteristics, likely aetiology and management of the study population.

	Traumatic (*n* = 10)	Spontaneous (*n* = 36)	Total (*n* = 46)	*p*-value
Type of ICH (%)				
Subdural	8 (80.0%)	2 (5.6%)	10 (21.7%)	<0.001
Intraventricular	0	1 (2.8%)	1 (2.2%)	
Intraparenchymal	0	9 (25.0%)	9 (19.6%)	
Mixed	2 (20.0%)	24 (66.7%)	26 (56.5%)	
Radiological prediction of likely underlying aetiology (%)				
Hypertensive vasculopathy	0	28 (77.8%)	28 (60.9%)	<0.001
Cerebral Amyloid Angiopathy	0	4 (11.1%)	4 (8.7%)	
Traumatic	10 (100%)	0.0	10 (21.7%)	
Unknown		4 (11.1%)	4 (8.7%)	
Management (%)				
Conservative	9 (90.0%)	36 (100.0%)	45 (97.8%)	0.1
Neurosurgical	1 (10.0%)	0	1 (2.2%)	

HAS-BLED scores between the traumatic and spontaneous ICH cohorts were also comparable. Although the median annual bleeding risk and patient-specific OAC was identical (2.8 [2.1–3.8] % in the traumatic ICH cohort, and 2.8 [1.9–4.1] % in the spontaneous ICH cohort), the median net clinical benefit was slightly higher for the traumatic ICH cohort compared to the spontaneous ICH cohort (3.1 vs. 2.4 strokes prevented per major bleed caused). According to SPARCtool, 69.6% (*n* = 32) of the study population were inappropriately anticoagulated, where the annual bleeding risk outweighed the annual thromboembolism risk for the patient-specific OAC, and the main modifiable factor is the presence of hypertension. When stratified according to type of ICH, more were inappropriately anticoagulated according to SPARCtool in the spontaneous cohort compared to the traumatic cohort (75% vs. 50%, respectively).

The significant *p*-value for aetiology is expected and should not be overinterpreted, as the aetiological definitions are intrinsically linked to whether the haemorrhage was spontaneous or traumatic. This makes the association partly circular. Therefore, the table is useful descriptively, but the p-value does not add meaningful inferential value (Table 2).

There were no statistically significant differences in baseline characteristics, frailty, or anticoagulation profiles between the traumatic and spontaneous ICH groups (*p* > 0.05).

## 4 Discussion

The annual rate of ICH in AF patients on OAC is approximately 1%. It is associated with a high mortality and acts as a major obstructing factor to the clinical decision of starting OAC therapy (Lopes et al., 2017; Hart et al., 2012; Hankey et al., 2014).

Our study demonstrated a skew of distribution in the type of ICH to spontaneous rather than traumatic, which is replicated in other studies (Lopes et al., 2017). A greater proportion of intraparenchymal or mixed bleeding were found in spontaneous ICH, opposed to subdural bleeding in those with a traumatic cause. Importantly, the median CFS score was 5 for both groups, which correlates with patients who are living with “mild frailty.” There was also no evidence that these were patients who were “frequent fallers” or had suspicion of significant mobility issues. Rather, our cohort demonstrated a high prevalence of concomitant modifiable risk factors—most notably, hypertension.

Within the spontaneous ICH group, 86.1% had an underlying diagnosis of hypertension, and the majority (28/36, 77.8%) of those patients had radiological evidence of hypertensive vasculopathy as the likely reason for their ICH. There are two key points that can be gleaned. First, hypertensive vasculopathy as a direct consequence of poorly controlled hypertension over time may be associated with spontaneous ICH in AF patients. Second, the identification of poorly managed hypertension in our cohort likely provides a piece to a larger issue—the management of all modifiable risk factors for AF must be improved.

The importance of OAC over the risk of major bleeding is re-emphasised in the updated ESC AF task force guideline, where the decision to anticoagulate is supported by removing the sex category as a risk factor to form the CHADS-VA risk score and removing bleed-risk-scoring such as HAS-BLED altogether (Van Gelder et al., 2024). It also strongly highlights the importance of managing risk factors, providing a strict blood pressure target range, as poor blood pressure control is also independently related to haematoma expansion, ventricular extension, and poor outcomes independent of OAC use in studies (Qureshi et al., 2010). In line with recent studies highlighting hypertension as a major cause of ICH-related mortality, our findings demonstrate that a large burden of ICH-related mortality is related to significant modifiable risk factors, predominantly hypertension in our study population (Zeng et al., 2023).

There is growing evidence suggesting the importance of OAC use in ICH survivors, where ICH in itself has been identified as an independent predictor of thromboembolic events among AF patients (Friberg et al., 2012). The optimal time to restart anticoagulation was also suggested to be as early as 7–8 weeks following the first bleeding event (Pennlert et al., 2017). However, these decisions are frequently influenced by perceived bleeding risks. In this context, our findings provide important insight that within this fatal ICH cohort, spontaneous haemorrhage associated with hypertensive vasculopathy was numerically more common than trauma-related ICH, despite comparable frailty and limited evidence of recurrent falls. While causality cannot be inferred, these observations highlight the need to consider chronic, potentially modifiable vascular risk factors alongside anticoagulation status when balancing bleeding and thromboembolic risk.

There are several limitations to this study. This is a retrospective analysis of fatal ICH cases in a single centre. While the sample size is modest, and results may not be generalisable globally, they reflect real-life experience in a large hyperacute stroke unit in the United Kingdom. Additionally, we are unable to comment on the effect of different types, durations, and adherence of OAC therapy on ICH-related mortality. Further details on exact blood pressure management and medication adherence would also provide further value, as it would allow further distinction and analysis of the temporal relationship between blood pressure control and ICH mortality. Other factors such as medical issues contributing to the increased falls risk could not be fully accounted for.

## 5 Conclusion

Hypertension is not only a major substrate of AF incidence but a cause of additional morbidity and mortality. Within our cohort, hypertensive vasculopathy was a significant underlying aetiology of spontaneous ICH-related mortality. Falls and trauma causing ICH in anticoagulated patients with AF may be overestimated. In line with the recently updated ESC guideline, blood pressure control should take priority in the management of AF to reduce major bleeding risk. Hypertension is an important modifiable factor that, when managed inadequately, can lead to consequential ICH-related mortality in anticoagulated patients with AF. Our findings emphasise and support early identification and stringent management of hypertension in the overall management of AF.

## Data Availability

The original contributions presented in the study are included in the article/Supplementary material, further inquiries can be directed to the corresponding author.

## References

[ref1] de Almeida J. P. H. C. Martinho A. S. Girão A. Barreiro I. Milner J. Ferreira M. J. V. . (2020). Novel anticoagulants in an older and frail population with atrial fibrillation: the effect of inappropriate dosing on clinical outcomes. Eur. Geriatr. Med. 11, 813–820. doi: 10.1007/s41999-020-00343-w 32557249

[ref2] Friberg L. Rosenqvist M. Lip G. Y. (2012). Evaluation of risk stratification schemes for ischaemic stroke and bleeding in 182 678 patients with atrial fibrillation: the Swedish atrial fibrillation cohort study. Eur. Heart J. 33, 1500–1510. doi: 10.1093/eurheartj/ehr488, 22246443

[ref3] Gage B. F. Birman-Deych E. Kerzner R. Radford M. J. Nilasena D. S. Rich M. W. (2005). Incidence of intracranial hemorrhage in patients with atrial fibrillation who are prone to fall. Am. J. Med. 118, 612–617. doi: 10.1016/j.amjmed.2005.02.022, 15922692

[ref4] Grymonprez M. Steurbaut S. De Backer T. L. Petrovic M. Lahousse L. (2020). Effectiveness and safety of Oral anticoagulants in older patients with atrial fibrillation: a systematic review and Meta-analysis. Front. Pharmacol. 11:583311. doi: 10.3389/fphar.2020.583311, 33013422 PMC7509201

[ref5] Hankey G. J. Stevens S. R. Piccini J. P. Lokhnygina Y. Mahaffey K. W. Halperin J. L. . (2014). Intracranial hemorrhage among patients with atrial fibrillation anticoagulated with warfarin or rivaroxaban: the rivaroxaban once daily, oral, direct factor Xa inhibition compared with vitamin K antagonism for prevention of stroke and embolism trial in atrial fibrillation. Stroke 45, 1304–1312. doi: 10.1161/STROKEAHA.113.004506, 24743444

[ref6] Hart R. G. Diener H. C. Yang S. Connolly S. J. Wallentin L. Reilly P. A. . (2012). Intracranial hemorrhage in atrial fibrillation patients during anticoagulation with warfarin or dabigatran: the RE-LY trial. Stroke 43, 1511–1517. doi: 10.1161/STROKEAHA.112.650614, 22492518

[ref7] Kelly D. M. Rothwell P. M. (2020). Blood pressure and the brain: the neurology of hypertension. Pract. Neurol. 20, 100–108. doi: 10.1136/practneurol-2019-002269, 31558584

[ref8] Kornej J. Börschel C. S. Benjamin E. J. Schnabel R. B. (2020). Epidemiology of atrial fibrillation in the 21st century: novel methods and new insights. Circ. Res. 127, 4–20. doi: 10.1161/CIRCRESAHA.120.316340, 32716709 PMC7577553

[ref9] Lopes R. D. Guimarães P. O. Kolls B. J. Wojdyla D. M. Bushnell C. D. Hanna M. . (2017). Intracranial hemorrhage in patients with atrial fibrillation receiving anticoagulation therapy. Blood 129, 2980–2987. doi: 10.1182/blood-2016-08-731638, 28356246

[ref10] Madhavan M. Holmes D. N. Piccini J. P. Ansell J. E. Fonarow G. C. Hylek E. M. . (2019). Association of frailty and cognitive impairment with benefits of oral anticoagulation in patients with atrial fibrillation. Am. Heart J. 211, 77–89. doi: 10.1016/j.ahj.2019.01.005, 30901602

[ref11] Man-Son-Hing M. Nichol G. Lau A. Laupacis A. (1999). Choosing antithrombotic therapy for elderly patients with atrial fibrillation who are at risk for falls. Arch. Intern. Med. 159, 677–685. doi: 10.1001/archinte.159.7.677, 10218746

[ref12] Miyasaka Y. Barnes M. E. Gersh B. J. Cha S. S. Bailey K. R. Abhayaratna W. P. . (2006). Secular trends in incidence of atrial fibrillation in Olmsted County, Minnesota, 1980 to 2000, and implications on the projections for future prevalence. Circulation 114, 119–125. doi: 10.1161/CIRCULATIONAHA.105.595140, 16818816

[ref13] Nabauer M. Gerth A. Limbourg T. Schneider S. Oeff M. Kirchhof P. . (2009). The registry of the German competence NETwork on atrial fibrillation: patient characteristics and initial management. Europace 11, 423–434. doi: 10.1093/europace/eun369, 19153087 PMC2659602

[ref14] Pennlert J. Overholser R. Asplund K. Carlberg B. Van Rompaye B. Wiklund P. G. . (2017). Optimal timing of anticoagulant treatment after intracerebral hemorrhage in patients with atrial fibrillation. Stroke 48, 314–320. doi: 10.1161/STROKEAHA.116.014643, 27999135

[ref15] Pinho-Gomes A. C. Azevedo L. Copland E. Canoy D. Nazarzadeh M. Ramakrishnan R. . (2021). Blood pressure-lowering treatment for the prevention of cardiovascular events in patients with atrial fibrillation: an individual participant data meta-analysis. PLoS Med. 18:e1003599. doi: 10.1371/journal.pmed.1003599, 34061831 PMC8168843

[ref16] Qureshi A. I. Palesch Y. Y. Martin R. Novitzke J. Cruz-Flores S. Ehtisham A. . (2010). Effect of systolic blood pressure reduction on hematoma expansion, perihematomal edema, and 3-month outcome among patients with intracerebral hemorrhage: results from the antihypertensive treatment of acute cerebral hemorrhage study. Arch. Neurol. 67, 570–576. doi: 10.1001/archneurol.2010.61, 20457956 PMC5562043

[ref17] Van Gelder I. C. Rienstra M. Bunting K. V. Casado-Arroyo R. Caso V. Crijns H. J. G. M. . (2024). 2024 ESC guidelines for the management of atrial fibrillation developed in collaboration with the European Association for Cardio-Thoracic Surgery (EACTS). Eur. Heart J. 45, 3314–3414. doi: 10.1093/eurheartj/ehae176, 39210723

[ref18] Wei W. Rasu R. S. Hernández-Muñoz J. J. Flores R. J. Rianon N. J. Hernández-Vizcarrondo G. A. . (2021). Impact of fall risk and direct Oral anticoagulant treatment on quality-adjusted life-years in older adults with atrial fibrillation: a Markov decision analysis. Drugs Aging 38, 713–723. doi: 10.1007/s40266-021-00870-6, 34235644

[ref19] Wolf P. A. Abbott R. D. Kannel W. B. (1991). Atrial fibrillation as an independent risk factor for stroke: the Framingham study. Stroke 22, 983–988. doi: 10.1161/01.STR.22.8.983, 1866765

[ref20] Zeng Z. Chen J. Qian J. Ma F. Lv M. Zhang J. (2023). Risk factors for anticoagulant-associated intracranial hemorrhage: a systematic review and Meta-analysis. Neurocrit. Care. 38, 812–820. https://doi:10.1007/s12028-022-01671-4. doi: 10.1007/s12028-022-01671-4, 36670269

