# Supplementary material for: Association between poor blood pressure control and intracranial haemorrhage-related mortality in patients with atrial fibrillation receiving oral anticoagulation: a retrospective observational cohort study
Source: Front Stroke. 2026 Aug 14;5:1840317. doi: 10.3389/fstro.2026.1840317 (PMC13521787; doi:10.3389/fstro.2026.1840317)
Supplement: Supplementary file 1 [file Data_Sheet_1.docx]

**Supplementary figures**

Supplementary Figure 1a – Example of Traumatic Subdural haemorrhage and associated features in study population. A large subdural haematoma is seen lining the left tentorium and extending over and around the left cerebrum.

Supplementary Figure 1b: Large posterior parietal midline scalp haematoma (same patient as Supplementary Figure 1a.)

Supplementary Figure 2 - Example of Spontaneous Intracerebral haemorrhage – likely Cerebral amyloid angiopathy-related in the study population. A large volume intraparenchymal bleed is seen in the left temporal-occipital lobe with significant oedema and midline shift. There is presence of sulcal blood.

Supplementary Figure 3 – Example of Spontaneous Intracerebral haemorrhage likely hypertensive vasculopathy-related in the study population. An area of hyperdensity is seen in the left capsular region with evidence of right hemispheric frontal lobe encephalomalacia and cerebral small vessel disease.
